# Hepatitis B viral load and risk for liver cirrhosis and hepatocellular carcinoma in The Gambia, West Africa

**DOI:** 10.1111/j.1365-2893.2009.01168.x

**Published:** 2010-02

**Authors:** M E Mendy, T Welzel, O A Lesi, P Hainaut, A J Hall, M H Kuniholm, S McConkey, J J Goedert, S Kaye, S Rowland-Jones, H Whittle, G D Kirk

**Affiliations:** 1 Viral Diseases Programe, Medical Research CouncilBanjul, The Gambia; 2 Division of Cancer Epidemiology and Genetics, National Cancer InstituteRockville, MD, USA; 3 Lagos University Teaching HospitalLagos, Nigeria; 4 International Agency for Research on CancerLyon, France; 5 London School of Hygiene and Tropical MedicineLondon; England; 6 Department of Epidemiology, Johns Hopkins Bloomberg School of Public HealthBaltimore, MD, USA; 7 Royal College of Surgeons in IrelandDublin, Ireland; 8 Imperial CollegeLondon, UK

**Keywords:** Africa, cirrhosis, HBV, HCC, viral load

## Abstract

The main objectives of this study were to define the occurrence and levels of hepatitis B virus (HBV) DNA in asymptomatic HBV carriers, cirrhosis patients and hepatocellular carcinoma (HCC) cases from The Gambia, and to evaluate the risk for cirrhosis or HCC associated with HBV viremia. We used sensitive real-time quantitative PCR assays to measure HBV DNA in samples from a case–control study consisting of 60 asymptomatic HBV carriers, 53 cirrhotic patients and 129 HCC cases. Logistic regression was used to estimate the risks of cirrhosis and HCC associated with HBV-DNA levels and HBV e antigenemia (HBeAg) detection (a surrogate marker for viral replication). Detectable HBV viremia and HBeAg positivity were both significantly associated with cirrhosis (increasing risk by fourfold and 11-fold respectively) and with HCC (increasing risk by sixfold and threefold respectively). HBV-DNA levels were significantly higher in both HCC cases and cirrhotic patients compared to asymptomatic carriers (*P*<0.01 for both). High-level HBV DNA (>10 000 copies/mL) was strongly associated with both HCC and cirrhosis (17- and 39-fold increased risk). Lower level HBV viremia (200–10 000 copies/mL) conferred a significant risk of HCC, although the association with cirrhosis was not significant. In conclusion, we find that high HBV-DNA levels are strongly associated with the serious sequelae of HBV infection, independent of HBeAg status. While risk for cirrhosis and for HCC notably increases at HBV-DNA levels ≥10 000 copies/mL, low-level viremia was also associated with significant risk for HCC.

## Introduction

Hepatitis B virus (HBV) is the leading cause of hepatocellular carcinoma (HCC) worldwide, particularly in Asia and sub-Saharan Africa [1]. In these endemic regions, HBV infection is generally acquired at birth or in early childhood with up to 10–20% of the adult population persistently expressing HBV surface antigen (HBsAg, a serologic marker of chronic infection) [2]. Persons with HBsAg expression are at very high risk of developing the chronic sequelae of HBV infection including liver cirrhosis and HCC. However, despite the very high attributable fraction and relative risk related to HBsAg positivity, only a minority of chronically infected persons will develop cirrhosis or HCC. The role of environmental or behavioural factors (e.g., aflatoxin exposure, alcohol use) and of host genetic factors have been documented to modify individual risk for HCC [3–5]. In addition, growing evidence indicates that differences in HBV outcomes may be closely associated with viral characteristics, including replication status and genetic variation of HBV [3,6,7]. Recent studies conducted in large Asian cohorts indicate that HBV-DNA levels strongly predicted development of HBV-related cirrhosis and HCC [8–10]. In contrast to Asia, there are only limited data on HBV-DNA detection and quantification associated with liver disease outcomes among persons from sub-Saharan Africa.

Hepatitis B virus infection is endemic in The Gambia in West Africa with a 15–20% prevalence among adults [11,12]. HCC is the most common cause of cancer and one of the leading causes of death among Gambians [13]; HBV accounts for around two-thirds of HCC cases [11]. Using hybrizidation assays, we previously reported that high-titre serum HBV DNA in adult HBV chronic carriers was correlated with duration of HBV carriage and HBeAg positivity [14]. However, HBV hybridization assays have limited sensitivity and in response, we recently validated a sensitive quantitative real-time polymerase chain reaction assay for measuring serum HBV DNA among Gambian HBV carriers [15]. In the current study, we apply this assay to measure HBV-DNA levels in a well-characterized Gambian study population that includes asymptomatic HBV carriers and persons with HBV-related cirrhosis or HCC. We hypothesized that a dose–response association of HBV-DNA level with advanced liver disease would be observed. Thus, our primary objectives were to define the occurrence and levels of HBV viremia and examine their associations with cirrhosis and HCC in an African population.

## Materials and methods

### Study participants

Our study population included all HBsAg-positive persons that had been prospectively recruited into the Gambia Liver Cancer Study (GLCS), as described previously [11]. Briefly, participants were recruited from September 1997 through January 2001 through liver disease referral clinics at three tertiary hospital sites (Royal Victoria Hospital, Banjul; Medical Research Council Hospital, Fajara; and Bansang Hospital, Bansang). Incident cases of cirrhosis or of HCC were identified from among patients with suspected liver disease referred by local physicians or identified through active surveillance of the wards and clinics by GLCS field staff. All participants were administered a standardized questionnaire and underwent a clinical examination and venipuncture; persons with liver disease had a standardized ultrasound examination performed. HCC was diagnosed either by pathology or by a combination of ultrasonographic evidence of space-occupying lesions and a serum α-fetoprotein (AFP) level of ≥100 ng/mL. Among individuals without pathologic or ultrasonographic evidence of HCC, cirrhosis was diagnosed using an ultrasound scoring system previously validated to liver histology among HBV-infected persons [10,16,17]. The quantitative cirrhosis score is derived from the ultrasonographic evaluation of the liver surface, liver parenchyma, caliber of intrahepatic blood vessels and spleen size; scores of ≥7 were used to define cirrhosis [18]. Asymptomatic HBV carriers were identified from among control participants without clinical evidence of liver disease recruited from the outpatient general medical clinics of the same hospital sites. Antiviral treatment active against HBV was not available to study participants. Local and international scientific and ethical review committees approved the study protocol, and informed consent was obtained from each participant.

### Laboratory testing

Following processing within 24 h, blood specimens were stored at either −20 or −70 °C depending on the planned testing. AFP was detected and quantified by standard radiometric assay methods (DiaSorin SA, Sallugia, Italy). Aspartate aminotransferase (AST), alanine aminotransferase (ALT) and total bilirubin levels were determined on clinical samples using Roche COBAS MIRA Chemistry Analyzer. HBsAg was determined by reverse passive haemagglutination assay (Murex Diagnostics Limited, Dartford, UK) with radioimmunoassay testing of negative samples (Sorin Biomedica Diagnostics, Vercelli, Italy). Participants positive for HBsAg were tested for HBV e antigenemia (HBeAg) as a surrogate marker of active replication using a radioimmunoassay kit (DiaSorin). HBV-DNA detection and quantification were performed as described previously [15]. Briefly, quantitative real-time PCR was carried out using commercial SYBR-Green reaction mix (Qiagen, Hilden, Germany) and primers specific to the S gene. The primer sequences were 5′ GTG TCT GCG GCG TTT TAT CA (sense) and 5′ GAC AAA CGG GCA ACA TAC CTT (antisense) designed to amplify a 98 base pair product from positions 379 to 476 of the HBV genome. Thermal cycling was performed in an ABI 5700 sequence detection system (PE Applied Biosystems, Warrington, UK). HBV-DNA concentration was calculated from a four point standard curve [1.5 × 10^8^, 1.5 × 10^6^, 1.5 × 10^4^ and 1.5 × 10^2^ copies/mL]. Calibration of this standard was confirmed by comparison with an international HBV-DNA standard (97/746; NIBSC, Potters Bar, UK). The detection limit of the RT-PCR assay was 2.6 × 10^2^ DNA copies per mL [15], although the qualitative limit of detection for the assay was established as 2.0 × 10^2^ DNA copies per mL. Samples testing above the standard curve were re-assayed at a dilution of 1:100. The assay was 100% specific when tested against 10 HBV negative sera and intra- and inter-assay comparisons demonstrated acceptable reproducibility [15].

### Statistical analysis

We calculated descriptive statistics for demographic and clinical variables by study group. Geometric mean (GM) and median HBV-DNA levels were determined based on observed value for participants with quantifiable HBV DNA (≥260 copies/mL) and an estimate of 200 copies/mL for participants with non-quantifiable but qualitatively detectable HBV DNA (200–259 copies/mL). We used chi-square, Fisher’s exact, unpaired *t* and unbalanced analysis of variance tests to evaluate differences among and between the three study groups. We used unadjusted and adjusted logistic regression models to evaluate the impact of HBeAg positivity, HBV-DNA detection status and HBV-DNA level on risk of cirrhosis or of HCC compared with control individuals. Based on prior analysis [11], we included age, gender, recruitment site and date, education and household floor type in adjusted models. Education and household floor type were included in each analysis as markers of socioeconomic status. Participants’ tobacco, alcohol consumption and the presence of antibodies against hepatitis C virus did not qualitatively change any effect estimates, and were thus excluded from the final models. Three individuals with incomplete ascertainment of educational status were excluded from the analyses of risk for cirrhosis and HCC. All analyses were conducted using SAS 9.1 (SAS Institute, Cary, NC, USA).

## Results

### Characteristics of study participants

Demographic and laboratory marker characteristics of the 60 asymptomatic HBV carriers, 53 HBV-related cirrhotic patients and 129 HBV-related HCC cases are presented in Table 1. All three groups were majority male with gender ratios ranging from 3.3 to 5.3 males per female. The majority of participants were under 45 years of age with minimal differences in this proportion by study group (asymptomatic carriers, 61%; cirrhosis cases, 63%; HCC cases, 59%). Study participants often lived in homes with earthen floors but most had received at least some formal schooling. Recruitment site and socioeconomic status variables did not differ significantly between the three study groups. Cigarette smoking was relatively common in this population although alcohol consumption was not (data not shown); the proportions of smokers and drinkers did not vary significantly between the study groups. Elevated AST and bilirubin levels were seen more frequently in the cirrhosis and HCC case groups compared to asymptomatic carrier group (both *P*<0.01), while the occurrence of elevated ALT was infrequent and similar across the three groups.

**Table 1:** Characteristics of 242 HBV e antigenemia (HBeAg)-positive participants by study group

Variable	HBV Carriers (*n* = 60) N (%)	Cirrhosis cases (*n* = 53) N (%)	HCC cases (*n* = 129) N (%)
Demographic			
Male gender	49 (82)	41 (77)	108 (84)
Mean age ± SD (years)	41 ± 16	39 ± 12	42 ± 13
Site			
RVH	17 (28)	28 (53)	54 (42)
MRC	21 (3)	13 (25)	40 (31)
BSG	22 (37)	12 (23)	35 (27)
Education			
Ever school	56 (93)	44 (83)	103 (80)
None	4 (7)	9 (17)	23 (18)
Unknown	0 (0)	0 (0)	3 (2)
Earth floor			
Yes	34 (57)	21 (40)	50 (39)
No	26 (43)	32 (60)	79 (61)
Laboratory			
AST (IU/L)			
AST ≥ 45	8 (13)	30 (57)	95 (74)
Missing	1 (2)	2 (4)	11 (9)
ALT (IU/L)			
ALT ≥ 45	2 (3)	2 (4)	7 (5)
Missing	2 (3)	2 (4)	11 (9)
Bilirubin (mg/dL)			
Bilirubin ≥ 20	16 (27)	37 (70)	67 (52)
Missing	3 (5)	3 (6)	18 (14)

### HBV markers by study group and demographic variables

**Fig. 1:**
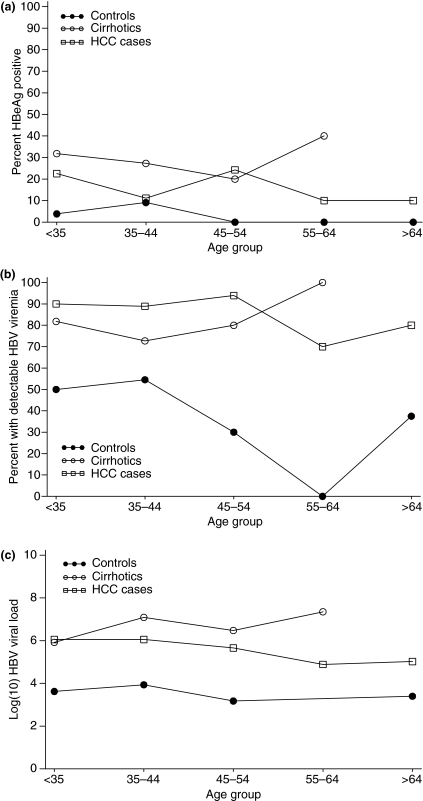
Age distribution of HBV e antigenemia (HBeAg) and hepatitis B virus (HBV)-DNA positivity (a and b) and HBV DNA load by age group (c).

Circulating HBeAg was detected in only 3% of asymptomatic carriers compared to 28% and 18% of cirrhosis and HCC cases respectively (Table 2). When examining the distribution of HBeAg postivity by age group (Fig. 1a), the proportion of cirrhosis and HCC cases who were HBeAg positive was higher than among asymptomatic carriers regardless of age; notably, we did not detect HBeAg among any asymptomatic HBV carriers over 44 years of age. Cirrhosis cases tended to have a higher proportion with HBeAg expression than HCC cases across most age strata.

**Table 2:** Hepatitis B virus (HBV) markers by study group

Variable	HBV Carriers (*n* = 60) N (%)	Cirrhosis Cases (*n* = 53) N (%)	HCC Cases (*n* = 129) N (%)
HBeAg+	2 (3.3)	15 (28)	23 (18)
HBV DNA+	24 (41.7)	43 (81.1)	114 (88.4)
HBV-DNA levels, Geometric Mean (Log_10_ Mean) in copies/mL	4143 (3.62)	2 887 109 (6.46)	634 794 (5.80)

Cirrhosis and HCC cases more commonly had detectable HBV viremia and higher viral load levels compared to asymptomatic HBV carriers (Table 2). Over 80% of cirrhosis and HCC cases had HBV-DNA detected compared to only 42% of asymptomatic carriers (*P*<0.01 for both comparisons). Among those participants with detectable HBV DNA, the GM of HBV-DNA levels among cirrhosis cases (6.46 log10 copies/mL) and HCC cases (5.80 log10 copies/mL) were more than 2 to 3 log10 higher, respectively, than observed among asymptomatic carriers (3.62 log10 copies/mL; *P*<0.01 for both comparisons). Similar relationships by study group were also observed when median HBV-DNA levels were examined with 1247 copies/mL, 6 398 892 copies/mL and 323 443 copies/mL for controls, cirrhosis and HCC cases, respectively. The findings of a lower proportion of viremia and lower viral load among asymptomatic carriers compared to subjects with advanced liver disease was consistently observed across all age groups (Fig. 1b, c).

Hepatocellular carcinoma cases 55 years of age and older tended to have a lower proportion of HBV-DNA detection (75%) compared to younger HCC cases (90.8% HBV DNA positive among those <55 years, *P*=0.058). In contrast, all cirrhosis cases 55 years of age and older had detectable HBV DNA compared to 79.2% among younger cirrhosis cases *P*=0.57). Overall, HBV-DNA levels among cirrhosis cases were higher (∼0.75 log10) than that of HCC cases (*P*=0.05). Further, this relationship of higher HBV-DNA levels among cirrhosis cases compared to HCC cases was observed within all age strata except for the youngest grouping of persons under 35 years of age (Fig. 1bc).

The distribution of HBV-DNA detection and viral loads within study groups, stratified by HBeAg status, are presented by age groupings and by gender in Table S1. HBeAg was perfectly predictive of HBV viremia as all HBeAg positive participants were also HBV-DNA positive. However, despite the commonly referenced use of HBeAg as a marker of HBV viral replication, 40% (23/58) of asymptomatic carriers, 74% (28/38) of cirrhosis cases and 86% (91/106) of HCC cases that were HBeAg negative had detectable HBV DNA (*P*=0.01). GM viral loads were significantly higher among HBeAg positive compared with HBeAg negative cirrhosis and HCC cases (*P*<0.01 for both). The two HBeAg positive asymptomatic carriers displayed much higher HBV-DNA levels compared with 58 HBeAg negative carriers; however, this comparison was limited by small numbers and was of borderline statistical significance (*P* = 0.05). Among HBeAg negative participants, higher HBV-DNA levels among cirrhosis cases compared with HCC cases were observed across all ages represented in the study.

Overall, male and female participants had similar proportions with detectable HBV DNA (76%*vs* 70%, respectively; *P*=0.42) and GM viral loads (478 319 *vs* 356 034, respectively; *P*=0.77). Gender-specific data on HBV-DNA detection and viremia stratified by study group and HBeAg status are presented in Table S1. Among HBeAg negative participants, males displayed higher HBV-DNA levels compared with females within each study group. In contrast, among HBeAg positive participants, males had lower HBV viral loads than females within each study group. However, these gender comparisons of HBV-DNA levels were limited by many strata having relatively small numbers, particularly for HBeAg positive women.

### HBeAg and HBV-DNA detection and risk of cirrhosis and HCC

After adjustment for age, gender, recruitment site, date and socioeconomic status, HBeAg positivity was associated with a significant 11.2-fold risk for cirrhosis and a 6.7-fold increased risk of HCC (Table 3). Adjusting for these same variables in a separate model, HBV viremia was significantly associated with both cirrhosis and HCC, increasing the risk by 5.7- and 12.1-fold respectively.

**Table 3:** HBV e antigenemia (HBeAg) and HBV-DNA detection and risk of cirrhosis and HCC

	Controls (*n* = 60)	Cirrhosis patients (*n* = 53)			HCC cases (*n* = 126)		
	Referent	OR	*OR	95% CI	OR	*OR	95% CI
HBeAg+	1.0	11.4	11.2	2.0, 61.5	6.1	6.7	1.5, 31.3
HBV DNA+	1.0	6.0	5.7	2.0, 15.9	11.2	12.1	5.3, 27.9
Combined model							
HBeAg+	1.0	6.2	6.2	1.1, 34.1	2.8	3.2	0.7, 15.3
HBV DNA+	1.0	4.3	4.1	1.4, 11.8	9.8	10.5	4.5, 24.5

OR, unadjusted odds ratio; *OR, odds ratio adjusted for age, gender, recruitment site and date, education and household floor type; HCC, hepatocellular carcinoma. 95% confidence interval presented is for the adjusted OR

In addition, we sought to estimate the independent risk for cirrhosis or HCC associated with HBeAg or HBV DNA, while accounting for detection of the other marker (Table 3). Even after adjustment for HBeAg status and other potential confounders, HBV viremia was significantly associated with both cirrhosis and HCC, increasing the risk by 4.1- and 10.5-fold respectively. While accounting for HBV-DNA detection, HBeAg positivity was associated with a significant 6.2-fold risk for cirrhosis and a 3.2-fold, but non-significant, increased risk of HCC.

### HBV-DNA levels and risk of HCC and cirrhosis

Adjusting for age, gender, recruitment site and date and socioeconomic status (Table 4), low-level HBV viremia (200–10 000 copies/mL) conferred a significant risk of HCC, but was not associated with cirrhosis compared to asymptomatic carriers. In contrast, high-level HBV viremia (>10 000 copies/mL) was strongly associated with both HCC and cirrhosis, conferring significant 17.3- and 38.8-fold increased risks of cirrhosis and HCC respectively.

**Table 4:** Hepatitis B virus (HBV)-DNA levels and risk of cirrhosis and HCC

	Controls (*n* = 60)	Cirrhosis cases (*n* = 53)			HCC cases (*n* = 126)		
HBV DNA (copies/mL)	Referent	OR	*OR	95% CI	OR	*OR	95% CI
200–10 000	1.0	1.2	1.0	0.3, 3.9	3.1	3.1	1.2, 8.2
>10 000	1.0	18.5	17.3	4.2, 71.2	32.1	38.8	12.1, 124.5

OR, unadjusted odds ratio; *OR, odds ratio adjusted for age, gender, recruitment site and date, education and household floor type and for HBeAg status; HCC, hepatocellular carcinoma. 95% confidence interval presented is for the adjusted OR.

## Discussion

In the current study, we present evidence that detection and quantification of HBV DNA among chronically infected persons provides valuable information for estimating risk for both cirrhosis and HCC. Our findings extend the utility of HBV-DNA measurements for predicting cirrhosis or HCC risk to include HBV-infected persons from sub-Saharan Africa. Further, despite the well-recognized difference in circulating HBV genotypes and the prevalence of viral mutations between Asian and African populations, we provide additional support that the threshold of 10 000 copies/mL of HBV DNA is important in the decision of antiviral treatment candidacy. However, our data raise further questions regarding the HCC risk associated with detectable HBV DNA at levels <10 000 copies/mL, and as such, appropriate management of persons with lower level HBV replication.

The recognition that relatively few chronic HBsAg-positive carriers manifest complications of HBV, the appreciation that productive viral replication commonly occurs in the absence of HBeAg, and the need for determining eligibility and for monitoring anti-HBV therapy all highlight the need for improved HBV biomarkers. Our current study applied a previously validated in-house RT-PCR assay to measure HBV-DNA levels among well-characterized participants in the Gambia Liver Cancer Study [11,15]. Importantly, all risk estimates associated with HBV-DNA measurements were in comparison with chronically infected, but asymptomatic persons expressing HBsAg of similar age to the cases. Our application of sensitive real-time PCR methods could detect low levels of HBV viremia. Simple categorization of HBV viremia as present was associated with a sixfold and a 12-fold increased risk for cirrhosis or HCC respectively. Further, we identified a dose–response increase in HCC risk with increasing levels of HBV DNA, up to a 39-fold risk associated with levels >10 000 copies/mL.

Large-scale cohorts with lengthy follow-up among Taiwanese HBV carriers have provided prospective data documenting an increased risk for cirrhosis and for HCC with increasing levels of HBV DNA [7,8,10]. Because of the differences in study design, risk estimates for cirrhosis and HCC with HBV viremia reported from our case–control study with HBV DNA measured at diagnosis, may not be directly comparable with estimates from the Asian cohort studies with HBV DNA measured on stored samples obtained 10–15 years prior to diagnosis [10]. If HBV-DNA levels decline over time to a greater degree among asymptomatic carriers compared with persons who develop advanced disease, risk estimates based on levels measured at diagnosis may overestimate risk. Conversely, if HBV DNA declines are accelerated among diseased compared to non-diseased, our risk estimates would be underestimated. Finally, if declines in HBV-DNA levels are comparable between persons who develop disease and similarly aged carrier controls, the risk estimates should also be comparable.

Only limited data are available to evaluate persistence of HBV-DNA levels. Chen and colleagues evaluated HBV-DNA levels from the visit just prior to HCC diagnosis on subjects with baseline HBV-DNA levels >10 000 copies/mL (median duration of interval follow-up was ∼10 years) [9]. HCC risk was most closely predicted by the proximate HBV-DNA measure; baseline levels of 10 000–99 000 copies/mL were not associated with significantly increased risk except among the 21% of persons with HBV-DNA levels increasing to >100 000 copies/mL. In subsequent detailed analysis of long-term HBV viral load measures in this cohort, the authors reported that while HCC cases had higher baseline viral loads, their HBV-DNA levels decreased more rapidly during follow-up compared to non-cases [19]; this suggests that our case–control study risk estimates may in fact be conservative. Importantly, if the baseline HBV-DNA measure was greater than ∼10 000 copies/mL in the Taiwanese cohort, the probability of maintaining stable levels above this threshold was high. Their data suggest that persistently elevated HBV-DNA levels are most predictive of developing HCC and support our premise that these markers are durable and useful for estimating risk later in the disease process.

Despite a similar overall prevalence of HBV carriage, there are marked differences in HBV infection between Asian and African populations. While perinatal transmission is common in Asian populations, horizontal transmission among young children predominates in Africa [2]. Differences in circulating HBV genotypes, HBeAg persistence, host genetics and exposure to cofactors such as aflatoxin may also contribute to a differing course of HBV infection between Asians and Africans. With all these population differences, it is remarkable that our findings from The Gambia are largely consistent with the prior Asian studies.

To our knowledge, only one prior study estimated HCC risk related to HBV-DNA measurements in an African population. Among 14 HCC cases matched to 22 HBsAg positive controls identified in a cohort of males in the Senegalese Army, Tang and colleagues reported a 16-fold increased risk with HBV-DNA detection [20], comparable to our risk estimate of 12.1 (Table 3).

Consistent findings suggest that risk for HCC associated with HBV-DNA levels increases in a dose–response fashion, but it remains unclear at what lower limit HCC risk may be negligible. Determination of the ‘safe’ threshold of HBV-DNA levels where the development of HBV-related complications is unlikely is of great importance in determining appropriate screening and antiviral management guidelines.

Prior studies of HBV viremia have largely focused on HCC as the primary endpoint. Chen and colleagues reported risk for cirrhosis and HCC outcomes achieving statistical significance at ≥10 000 copies/mL [9,10]. In a large prospective cohort in Haimen City in China, significantly increased risk for HCC and cirrhosis mortality was not observed until ≥100 000 copies/mL [9]. Our data suggested that while lower level viremia (200–10 000 DNA copies/mL) was significantly associated with HCC, risk for cirrhosis did not increase until ≥10 000 copies/mL. Interestingly, we found that HBV-DNA levels appeared to be higher among cirrhotic compared to non-cirrhotic HCC. Although viral factors largely did not differ between HBV-related non-cirrhotic compared to cirrhotic HCC in a Taiwanese study, there was a borderline association of higher viral load in cirrhotic HCC [21]. Younger HCC patients with lower HBV viral loads are less likely to have coexistent cirrhosis compared to older HCC cases with higher viremia [22].

These epidemiologic data can suggest mechanisms that may be involved in the pathogenesis of HBV-related cirrhosis and HCC. Replicative HBV infection is the stimulus for host immune responses leading to the chronic process of hepatocyte destruction and regeneration with development of fibrosis and eventually cirrhosis. As such, it may be that a certain threshold level of HBV replication is required to lead to development of cirrhosis.

It remains unclear whether HBeAg plays a direct biological role or merely represents a long-recognized surrogate marker for HBV replication [23]. HBeAg postivity is strongly associated with increased risk for HCC [6,11] while HBeAg seroconversion is mostly accompanied by resolution of necroinflammatory liver changes and reduced or undetectable HBV-DNA levels [24,25]. In our study, while all HBeAg positive participants were HBV-DNA positive, less than half of HBeAg negative asymptomatic carriers had detectable viremia. However, only 15–25% of those with cirrhosis or HCC expressed HBeAg at diagnosis. The incremental value of HBeAg status to predict liver disease risk was assessed (Table 3); interestingly, risk estimates for both cirrhosis and for HCC related to HBeAg positivity were reduced by almost half after accounting for HBV viremia. Assessment of the role of HBeAg-negative variants in relation to HBV-DNA levels and disease outcomes will be key to fully understand the mechanisms involved.

Our study had several challenges. Despite being one of largest of the few studies of HBV DNA in HCC or cirrhosis from Africa, some of our analyses were limited by having small numbers. As discussed above, the cross-sectional nature of our study evaluates HBV-DNA levels at time of diagnosis so the disease process itself may impact our measured levels. While we are unable to equivocally state what long-term predictive value HBV-DNA levels have in estimating risk for HBV-related liver disease, the consistency of our findings to previous studies, especially those with testing near time of diagnosis, supports our ability to make strong inferences.

In conclusion, we find that HBV-DNA levels are strongly associated with the serious sequelae of HBV infection, independent of HBeAg status. While risk for cirrhosis and for HCC notably increases at HBV-DNA levels ≥10 000 copies/mL, low-level viremia was also associated with significant risk for HCC. Growing evidence suggests that suppression of HBV, even if only for a finite time period, may significantly reduce risk for developing HCC [26]. Further application of sensitive HBV-DNA assays to appropriate study populations should improve our ability to target HBV-infected persons at highest risk for disease progression and most appropriate for antiviral therapy. In recent years, the successful introduction and dissemination of antiviral therapy for HIV infection (commonly including drugs with anti-HBV efficacy) in sub-Saharan Africa raises expectations that resource-constrained HBV-endemic regions of the world may also improve access to appropriate HBV treatment. Clearly, randomized trials of HBV therapies in these affected regions, with longer term follow-up to determine reductions in development of cirrhosis or HCC, are needed.

## Statement of interest

Drs Mendy, Hall, Whittle and Kirk contributed to study design; Drs Kirk, Goedert, Hainaut, Rowland-Jones and Whittle obtained funding support; Drs Lesi and Kirk contributed to patient evaluation and clinical data collection; Drs Mendy, Hainaut, McConkey and Kaye contributed to laboratory analyses; Drs Mendy, Welzel, Kuniholm and Kirk contributed to analysis of data; Drs Mendy, Welzel, Kuniholm and Kirk contributed to manuscript preparation. All authors contributed to critical review and editing of the manuscript.

## Abbreviations:

ALT: alanine aminotransferase
AST: aspartate aminotransferase
GLCS: Gambia Liver Cancer Study
GM: geometric mean
HBeAg: HBV e antigenemia
HBsAg: HBV surface antigen
HBV: hepatitis B virus
HCC: hepatocellular carcinoma

## SUPPORTING INFORMATION

**Table S1.** HBV DNA detection and viral load among HCC cases, cirrhotics and controls stratified by HBeAg status
