# Supplementary material for: Hepatitis B viral load and risk for liver cirrhosis and hepatocellular carcinoma in The Gambia, West Africa
Source: J Viral Hepat. 2010 Feb;17(2):115–22. doi: 10.1111/j.1365-2893.2009.01168.x (PMC2817443; doi:10.1111/j.1365-2893.2009.01168.x)
Supplement: Supplementary file 1 [file jvh0017-0115-SD1.doc]

| **Supplementary Table 1.** HBV DNA detection and viral load among HCC cases, Cirrhotics and controls stratified by HBeAg status | | | | | | | | | | | | |
| --- | --- | --- | --- | --- | --- | --- | --- | --- | --- | --- | --- | --- |
|  | HBeAg positive (n=40) | | | | | | HBeAg negative (n=202) | | | | | |
|  | Controls (n=2) | | Cirrhosis Cases (n=15) | | HCC Cases (n=23) | | Controls (n=58) | | Cirrhosis Cases (n=38) | | HCC Cases (n=106) | |
|  | N (%) | GM* | N (%) | GM* | N (%) | GM* | N (%) | GM* | N (%) | GM* | N (%) | GM* |
| HBV DNA+ | 2 (100) | 52,400,712 | 15 (100) | 21,986,411 | 23 (100) | 16,377,779 | 23 (40) | 1,822 | 28 (74) | 973,035 | 91 (86) | 279,158 |
| Age group |  |  |  |  |  |  |  |  |  |  |  |  |
| < 35 | 1 (100) | 173,091,224 | 7 (100) | 10,669,204 | 9 (100) | 52,556,771 | 12 (48) | 1,730 | 11 (73) | 164,637 | 27 (87) | 314,880 |
| 35 – 44 | 1 (100) | 15,863,511 | 3 (100) | 35,928,953 | 4 (100) | 118,744,208 | 5 (50) | 1,919 | 5 (63) | 6,477,324 | 28 (88) | 583,279 |
| 45 – 54 | * | * | 3 (100) | 20,602,121 | 8 (100) | 3,080,410 | 3 (30) | 1,493 | 9 (75) | 1,580,724 | 23 (92) | 234,972 |
| 55 – 64 | * | * | 2 (100) | 145,767,505 | 1 (100) | 12,212 | 0 (0) | * | 3 (100) | 6,501,876 | 6 (67) | 104,897 |
| ≥ 65 | * | * | * | * | 1 (100) | 140,642,613 | 3 (38) | 2,514 | * | * | 7 (78) | 37,520 |
| Gender |  |  |  |  |  |  |  |  |  |  |  |  |
| Men | 2 (100) | 52,400,712 | 12 (100) | 12,923,422 | 19 (100) | 10,064,919 | 18 (38) | 2,053 | 23 (79) | 1,112,883 | 77 (87) | 331,915 |
| Women | * | * | 3 (100) | 184,188,098 | 4 (100) | 165,431,241 | 5 (46) | 1,188 | 5 (56) | 524,630 | 14 (83) | 107,739 |
| * GM = Geometric mean HBV viral load (copies/ml) | | | | | | | | | | | | |
